# All-optical active THz metasurfaces for ultrafast polarization switching and dynamic beam splitting

**DOI:** 10.1038/s41377-018-0024-y

**Published:** 2018-07-04

**Authors:** Longqing Cong, Yogesh Kumar Srivastava, Huifang Zhang, Xueqian Zhang, Jiaguang Han, Ranjan Singh

**Affiliations:** 1 Division of Physics and Applied Physics, School of Physical and Mathematical Sciences, Nanyang Technological University, 21 Nanyang Link, Singapore, 637371 Singapore; 2 Centre for Disruptive Photonic Technologies, The Photonics Institute, Nanyang Technological University, 50 Nanyang Avenue, Singapore, 639798 Singapore; 3 Center for Terahertz Waves and College of Precision Instrument and Optoelectronics Engineering, Key Laboratory of Optoelectronic Information Technology, Ministry of Education, Tianjin University, 300072 Tianjin, People’s Republic of China

## Abstract

Miniaturized ultrafast switchable optical components with an extremely compact size and a high-speed response will be the core of next-generation all-optical devices instead of traditional integrated circuits, which are approaching the bottleneck of Moore’s Law. Metasurfaces have emerged as fascinating subwavelength flat optical components and devices for light focusing and holography applications. However, these devices exhibit a severe limitation due to their natural passive response. Here we introduce an active hybrid metasurface integrated with patterned semiconductor inclusions for all-optical active control of terahertz waves. Ultrafast modulation of polarization states and the beam splitting ratio are experimentally demonstrated on a time scale of 667 ps. This scheme of hybrid metasurfaces could also be extended to the design of various free-space all-optical active devices, such as varifocal planar lenses, switchable vector beam generators, and components for holography in ultrafast imaging, display, and high-fidelity terahertz wireless communication systems.

## Active metasurfaces: Terahertz control

All-optical control and manipulation of terahertz waves is now possible thanks to the development of custom-designed, dynamic reconfigurable metasurfaces. Realized by Longqing Cong and coworkers from Nanyang Technological University in Singapore and Tianjin University in China, the metasurfaces enable ultrafast (sub-nanosecond) polarization switching and beam splitting. They consist of an array of miniature aluminum-coated silicon split-ring resonators on a sapphire substrate. Illumination with short pulses of an infrared “pump beam” causes charge carriers in the silicon patch of the microring to transition from the valence to the conduction band, temporarily changing the photoconductivity and thus switching the transmission of the metasurface between “off” and “on” states for a polarized terahertz wave. Such high-speed switching of polarization is expected to be useful for applications such as data-encoding and multiplexing in a terahertz communications system as well as holography.

## Introduction

The widely applied optical components are mostly developed from natural materials, utilizing their refractive or diffractive effects for applications in wave plates, lenses, gratings, and beam splitters. Although these components have exhibited excellent performance in display, imaging, and optical communication systems, their bulky size limits their integration into miniaturized and compact optical systems, and their passive responses fail to provide switchable features for more advanced optical applications. The rapid progress in metamaterial research has attracted much attention for developing functional metadevices with extraordinary properties enabled by the artificially designed building blocks^1–3^. However, the practical applications of these metadevices are still limited due to the complex fabrication techniques, material losses, and operation bandwidths. The emergence of planar metasurfaces has enabled promising applications for planar optical components and has opened up opportunities for unprecedented control of electromagnetic waves^4–9^. One of the most promising applications of metasurfaces that has been discovered recently is as coding metamaterials^10–17^. To date, most of the studies in manipulating light using metasurfaces focus on improving the operation efficiency^18–20^, solving aberrations^21, 22^, and broadening the bandwidth^23–25^. However, less attention has been devoted to investigating active routes for switchable or multifunctional metasurface devices whose configuration could be dynamically controlled using external stimuli^26–29^. Potential approaches have been reported for the dynamic modulation of metasurfaces^30^, such as by integrating varactor or PIN diodes^31, 32^, microelectromechanical system-based actuators^33, 34^, semiconductor materials (silicon, GaAs, and conducting oxide)^27, 35–40^, two-dimensional materials (graphene and perovskite)^29, 41, 42^, phase change materials (VO_2_ and GST)^26, 28, 43^, and nonlinear materials^44, 45^. All of these approaches have revealed excellent capabilities in modulating the responses of a metadevice via electrical, optical, or thermal stimuli, with their own respective merits and demerits. As an excellent and mature complementary metal–oxide–semiconductor-compatible material, silicon has manifested its special appeal in industrial devices for electronic integrated circuits, solar cells, and solid-state lasers. In particular, silicon-based hybrid metasurfaces have been demonstrated for the modulation of the Fano resonance^46^, electromagnetically induced transparency, and slow light behaviors^35^ due to the fabrication flexibility and excellent optical properties of silicon. However, there are very few reports of dynamic metadevices that could be used as multifunctional planar optical devices (see supplementary Table S1). In this work, we propose a scheme for a hybrid terahertz anisotropic resonator, which acts as a basic building block of a miniature all-optical active metasurface integrated with a patterned silicon epilayer. We experimentally demonstrate an ultrafast all-optical modulation of transmitted polarization states and dynamic beam splitting in a hybrid metal–silicon metamaterial resonator scheme.

## Materials and methods

### Hybrid circular split ring resonator (*h*-SRR)

**Fig. 1:**
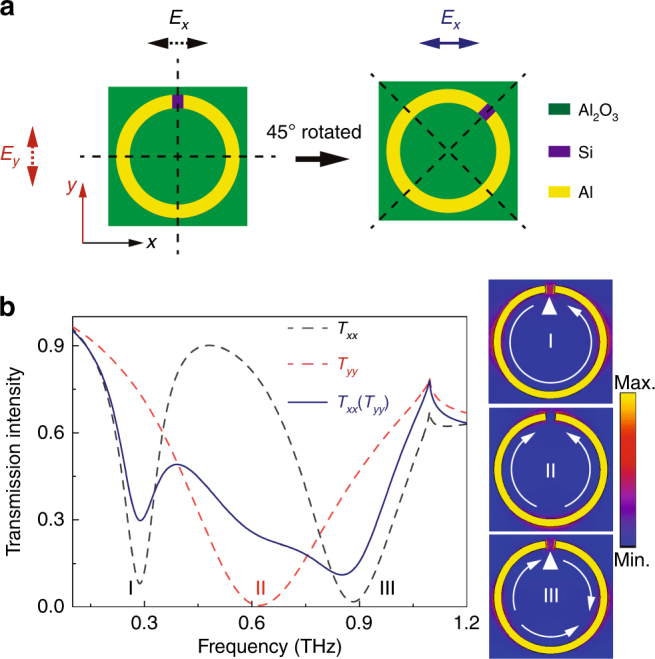
Anisotropic property of an *h*-SRR. **a** Schematic illustrations of an anisotropic *h*-SRR with different configurations relative to the incident electric field polarization. **b** Simulated transmission intensity spectra of the *h*-SRR resonator array. All three fundamental modes are simultaneously excited by rotating the *h*-SRR by 45° relative to the linear incident polarization (solid dark blue line). Intrinsic resonance modes (dashed lines) are induced by orthogonal polarization excitation along the optical axes of the resonator in the transmission spectra, with the respective surface current (solid-line arrows) and electric field (gradient color) distributions

A *h*-SRR on a square unit cell is adopted as a basic building block for the study of metasurface properties. In Fig. 1a, the green-color square is a sapphire (Al_2_O_3_) substrate, the purple part is a circular silicon (Si) ring on top of the sapphire, and the yellow-color split ring is made of aluminum on top of the silicon ring. The split gap in the metallic ring breaks the symmetry of the unit cell structure, which leads to different resonance behaviors for orthogonal polarization excitations, i.e., parallel (along the *y* axis) and perpendicular (along the *x* axis) to the mirror symmetric axis. As shown by the dashed lines in Fig. 1b, a typical inductive-capacitive (LC) resonance (mode I) and a third-order resonance (mode III) are induced by an *x*-polarized incidence, and a dipole resonance (mode II) is excited by a *y*-polarized incidence in the frequency range of interest. Relative to a linearly polarized incidence, an in-plane rotation of the resonator by 45° results in an excitation of all three fundamental resonance modes, which enables a broadened spectrum, as shown by the solid line in Fig. 1b. These three modes reveal discrete resonance behaviors and show different radiative properties that are observed via simulated surface currents and electric fields at the respective resonance frequencies. Figure 1b represents the resultant electric field concentration at the three resonance modes: a strong confinement is observed in the capacitive gap localized at modes I and III and a faint electric field is observed in the gap at mode II. Such a mode difference allows a distinct control of resonance behaviors and enables an active modulation of the anisotropy of the *h*-SRR by selectively damping modes I and III. Several promising active metadevices and ultrafast switches could be developed on the basis of the *h*-SRR.

### All-optical active modulation

**Fig. 2:**
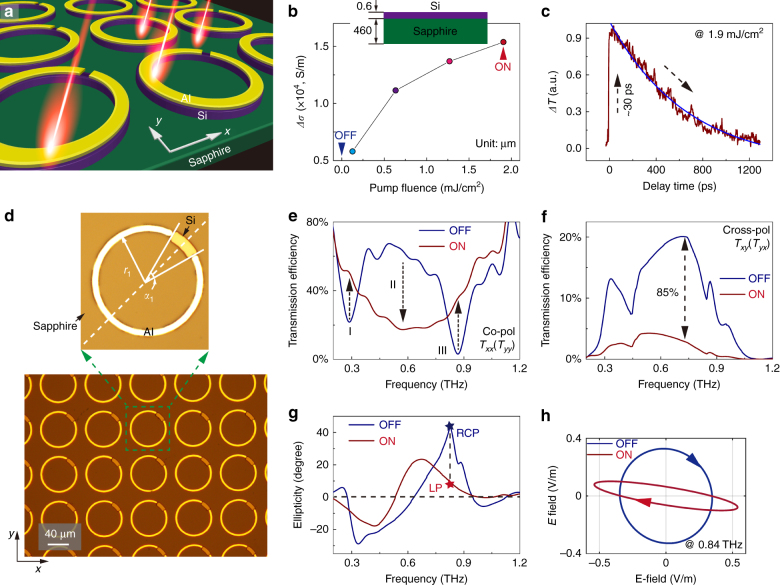
Active control of anisotropy in *h*-SRR metasurface. **a** Schematic illustration of the *h*-SRR metasurface being pumped by near-infrared femtosecond pulses. **b** Experimentally retrieved photoconductivity at various pump fluences for an unpatterned SOS substrate with a 0.6 μm thick silicon epilayer and a 460 μm thick sapphire substrate, and **c** the photocarrier excitation and relaxation dynamics of the silicon epilayer at a pump fluence of 1.9 mJ/cm^2^. **d** Microscopic images of the fabricated *h*-SRR sample array with a zoomed-in image showing the details of an individual resonator with geometrical parameters (in μm) *r*_1_ = 37, *α*_1_ = 10°, metal width = 5, and square period = 80. **e**, **f** Experimental demonstration of all-optical active control of the anisotropy in terms of mode switching in the co- and cross-polarized transmission spectra; the 85% modulation is on the basis of the OFF state spectrum. **g** Resultant ultrafast modulation of the output polarization state in terms of ellipticity, and **h** calculated two output polarization ellipses (RCP and LP) in the “ON” and “OFF” states on the basis of experimental data

When a thin semiconductor epitaxial layer (Si, bandgap ~1.1 eV) was incorporated in the *h*-SRR, the optical properties were actively controlled by an external optical pump (pulsed laser @ 800 nm, 1 kHz repetition rate, ~120 fs pulse width, and beam spot diameter *Ф* ~ 10 mm), as illustrated in Fig. 2a. The pump beam has a higher photon energy than the bandgap of the patterned silicon in order to pump the carriers from the valence band to the conduction band, which produces dynamic photoconductivity. A detailed study of the photoconductivity at different pump fluences was performed using an optical pump terahertz probe (OPTP) setup with unpatterned silicon on sapphire (SOS) substrate (see supplementary Note S1). Frequency-resolved photoconductivity ($\Delta \sigma \left( \omega \right)$, relative to the static conductivity without pump fluence) was obtained from $\Delta \sigma \left( \omega \right) = \frac{{\varepsilon _0c}}{d}\left( {n_{\mathrm{a}} + n_{\mathrm{b}}} \right)\frac{{ - \Delta E\left( \omega \right)}}{{E_0\left( \omega \right)}}$, where *ε*_0_ is the free space permittivity; *c* is the speed of light in free space; *d* is the silicon epitaxial layer thickness (600 nm); *n*_a_ and *n*_b_ are the refractive indices of the media on either side of the sample, i.e., air and sapphire; $E_0\left( \omega \right)$ is the transmission signal of the sample in the absence of photoexcitation; and $\Delta E\left( \omega \right)$ is the transmission difference ($\Delta E\left( \omega \right) = E_{\mathrm{p}}\left( \omega \right) - E_0\left( \omega \right)$) at different pump fluences ($E_{\mathrm{p}}\left( \omega \right)$). The experimentally obtained data is plotted in supplementary Figure S1, which shows a slight dispersion in the frequency band of interest (0.4–1.0 THz), and thus we consider a fluence-dependent photoconductivity at 0.7 THz for simplicity, as shown in Fig. 2b. We observe an enhanced photoconductivity of the silicon thin film from the static state (~160 S/m)^35^ to 15,369 S/m at a pump fluence of 1.9 mJ/cm^2^ (see supplementary Note S2). To show the switching behavior of the anisotropy in the *h*-SRR, we define two states: “OFF” representing the state when the photoexcitation is absent and “ON” representing a high fluence (1.9 mJ/cm^2^) photoexcited state. The switching speed of the device is determined by the accumulation and relaxation time of the photocarriers in the epitaxial silicon layer on the sapphire substrate, as shown in Fig. 2c. The maximum excitation of photocarriers occurs at approximately 30 ps after the pump pulse is incident on the sample at *t* *=* 0 ps, and the relaxation (recombination) dynamics is described by a monoexponential decay equation $\Delta T = 1.17 \cdot e^{ - (t - 30)/667} - 0.14$. The decay constant is determined to be 667 ps, which is adopted as the switching time of the hybrid metasurface since the resonances switch off and on at this time scale. Faster relaxation dynamics of silicon could be achieved by introducing defects or implanting ions into the intrinsic silicon that show two orders of magnitude faster switching time (see supplementary Figure S2). Another possible solution is by introducing doped high-mobility plasmonic materials (In-doped CdO) into the hybrid configuration via intraband pumping, for which the relaxation was reported to be on the femtosecond time scale^38^. We note that the intrinsic photoconductivity and relaxation dynamics of SOS substrates are identical to those in the operation of hybrid metadevices (see supplementary Figure S3 and Note S3).

The active control of the anisotropy in the *h*-SRR (see Fig. 2d) was experimentally probed using the OPTP setup (see supplementary Note S1). As shown in Fig. 2e, all three fundamental resonance modes were simultaneously induced in the co-polarized (*T*_*xx*_ or *T*_*yy*_) transmission spectrum in the OFF state of the *h*-SRR with a 45° orientation of the gap relative to the linearly polarized incidence. As a result of the anisotropy, there is a large magnitude of the cross-polarized component (*T*_*xy*_ or *T*_*yx*_) in a broad frequency regime, as shown in Fig. 2f. Notably, the two diagonal elements in the Jones matrix are identical to each other, i.e., *T*_*xx*_ = *T*_*yy*_ and *T*_*yx*_ = *T*_*xy*_ (see supplementary Figure S4 and Note S4), since the symmetric axis of the planar *h*-SRR lies along the diagonal line (dashed line of zoomed-in image in Fig. 2d). As the optical pump is switched on with a fluence of 1.9 mJ/cm^2^, the photoconductivity in the patterned silicon epilayer increases, especially in the segment of the split gap region. The highly conductive silicon segments thus selectively damp resonance modes I and III due to the strong confinement of the electric field in the split gap. Therefore, the isotropy (symmetry) of the *h*-SRR is restored. Such a modulation behavior is captured in the co- and cross-polarized transmission spectra, as shown in Fig. 2e, f, where the disappearance of modes I and III is observed compared to the OFF state spectrum. An active evolution from a multimode broadband spectrum in the OFF state to a pure dipole resonance mode in the ON state is realized by the *h*-SRR metasurface. Meanwhile, the restored isotropy due to the shorting of the capacitive gap of the *h*-SRR unit cell in the ON state also reflects the largely reduced magnitude of the cross-polarized component (a relative 85% modulation at 0.72 THz).

## Results and Discussion

### Ultrafast active polarization switch

One of the most straightforward results in the active modulation of the anisotropy is the tailoring of the output polarization states. The measured output polarization states that were characterized by ellipticity (see supplementary Note S5) are plotted in Fig. 2g. We clearly observe the contrast between the output polarization ellipses in a broad operation band for the ON and OFF states. Such an ultrafast polarization switch is visualized in Fig. 2h with typical polarization ellipses captured at 0.84 THz, where the largest polarization contrast is observed. Notably, a large polarization change from right-handed circular polarization (RCP, OFF state) to quasi-linear polarization (LP, ON state) is obtained, which exhibits a better polarization contrast compared to the recently reported results^38, 47^. The output polarization state can also be continuously modulated at different pump fluences (see supplementary Figure S5). Moreover, a stable response was observed for an *s*-polarized incidence at an oblique angle as large as 30° (see supplementary Figure S6). Such an all-optical polarization switch can be re-activated on a sub-nanosecond time scale determined by the photocarrier dynamics of the silicon epilayer, which is highly promising for ultrafast and high-fidelity polarization-division data encoding and multiplexing in ultrafast optical and terahertz communication systems.

### All-optical active beam splitter

A more interesting application on the basis of the *h*-SRR is an active metasurface for planar optical components, such as beam splitters^48, 49^, planar focal lens^21^, and holographic displays^18^. Since all of these applications are based on the two-dimensional logical phase distributions of the local resonators, we experimentally demonstrate an active beam splitter as a proof of concept using the all-optical active *h*-SRR resonator.

**Fig. 3:**
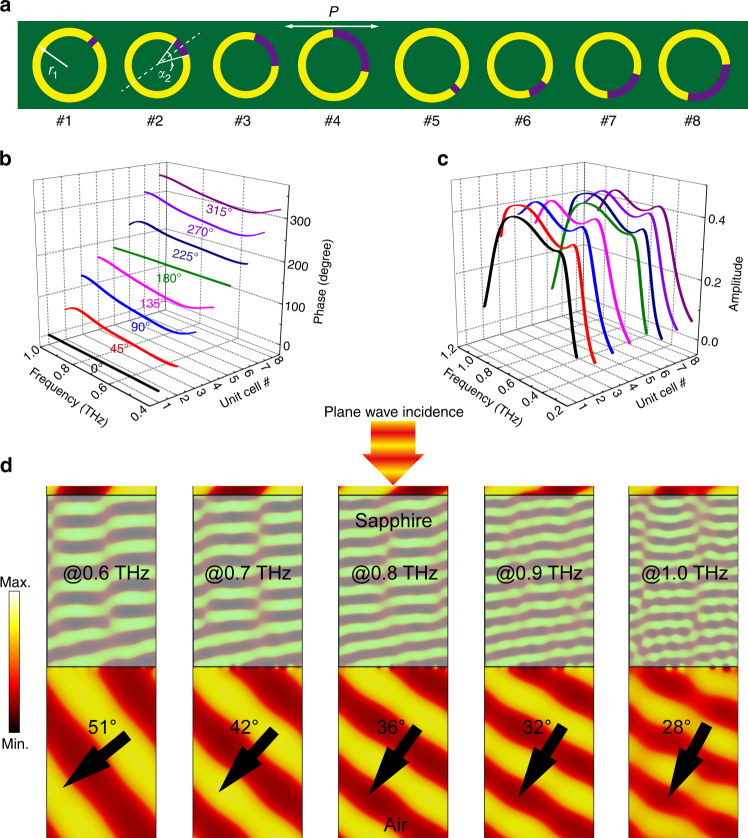
Wavefront engineering with *h*-SRRs. **a** Configurations of one supercell consisting of eight *h*-SRR unit cells for wavefront engineering. All the unit cells are designed with metal widths of 5 μm and periods *p* of 80 μm. The radii (*r*_n_) and gap splitting angles (*α*_n_) are tailored to obtain the relative phase response for unit cells #1 to #4 with *r*_1_ = 37, *α*_1_ = 10°; *r*_2_ = 34, *α*_2_ = 30°; *r*_3_ = 34, *α*_3_ = 80°; and *r*_4_ = 35, *α*_4_ = 110°. Unit cells #5 to #8 are identical to #1 to #4 but are rotated clockwise by 90°. **b**, **c** Simulated phase and amplitude spectra of cross-polarized component of each single *h*-SRR unit cell. **d** Simulated wavefront deflection of the cross-polarized component with plane wave incidence at different discrete frequencies ranging from 0.6 THz to 1.0 THz

To obtain the full coverage of 360° phase response of the local radiation, we carefully tailor the geometrical parameters of the *h*-SRR by varying its radius (*r*_n_) and arc angle (*α*_n_) that form the active split gap, where the subscript *n* is the series number of a single resonator. First, we obtain the radiative phase values of the cross-polarized components evenly covering the 180° range^2, 24^ from unit cells #1 to #4, as shown in Fig. 3a. With the broadband cross-polarized components of the anisotropic resonator in the OFF state, the corresponding phase values (using the phase response of unit cell #1 as a reference) also reveal a broadband behavior that is visualized in Fig. 3b. Second, by employing the Pancharatnam–Berry phase^50^, the phase coverage from 180° to 360° in unit cells #5 to #8 is achieved by rotating unit cells #1 to #4 by 90°, which introduces an extra 180° geometric phase. Unit cells #1 to #8 form a supercell that provides the extra momentum for cross-polarized transmitted light so that the wavefront is deflected from the metasurface normal with an angle of *θ*_crs-pol_ following the generalized Snell’s law equation: $\theta _{\mathrm{crs - pol}} = arc\sin \left( {\frac{{\lambda _0}}{{2\pi }}\frac{{d\varphi }}{{dy}}} \right)$, where $d\varphi$ and *dy* are the phase difference and geometrical distance between the neighboring unit cells, respectively, and *λ*_0_ is the wavelength of light. In addition to the phase response, the radiation amplitude of each unit cell determines the overall quality of the deflected wavefront and efficiency. The respective cross-polarized transmission amplitude is plotted in Fig. 3c, where we obtain an approximately constant amplitude for all eight unit cells in a broadband regime with an overall amplitude of approximately 0.4. Consequently, we could realize a high-quality polarized plane wave in a broadband range that is dispersed toward different angles relative to the metasurface normal following the generalized Snell’s law (Fig. 3d).

**Fig. 4:**
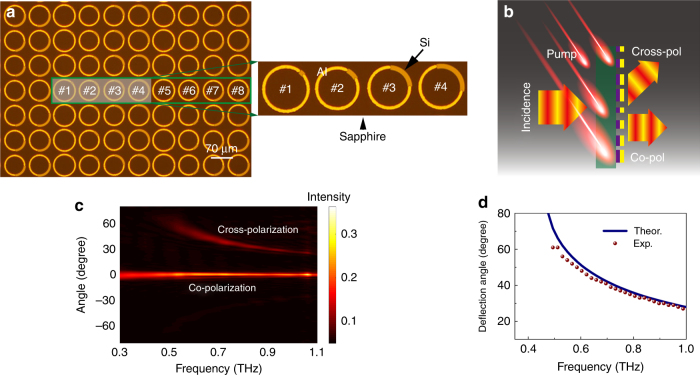
Experimental demonstration of dispersed wavefront deflection. **a** Microscopic images of a fabricated hybrid metasurface with a zoomed-in image. **b** Schematic diagram of the hybrid metasurface operating as an active polarizing beam splitter, and **c** the measured transmission intensity of the co- and cross-polarized components dispersed in angular space in a broadband range. **d** The measured deflection angle dispersion for the cross-polarized component following the theoretical model

Figure 4a shows the fabricated *h*-SRR supercell sample (see supplementary Note S1). Since the cross-polarized component deviates from the normal transmission of the co-polarized component (see supplementary Figure S7 and Note S6), the hybrid metasurface operates as a polarizing beam splitter in the static state, as illustrated in Fig. 4b. Angle-resolved measurements were performed using a set of fiber-based terahertz time-domain system (see supplementary Note S1) by sweeping the angle from −80° to +80° at an angular resolution of 1°. The experimental results are plotted in Fig. 4c. The normal direction is defined as 0°, where all the co-polarized transmission component is collected without dispersion and the cross-polarized component bends toward different positive angles depending on the frequencies such that dispersive polarizing beam splitting is realized in a broad terahertz band. The overall static transmission efficiencies for the co- and cross-polarized components are ~23% and ~15%, respectively, giving rise to a splitting ratio of ~60:40 for the normal and deflected beams. The measured dispersive components that are deflected at different angles obey the generalized Snell’s law equation (Fig. 4d), which also validates the measured results. The agreement between the experimental results and theoretical prediction is excellent except for the slight deviation observed at lower frequencies, which is mainly due to the large deflection angles and low signal intensity.

**Fig. 5:**
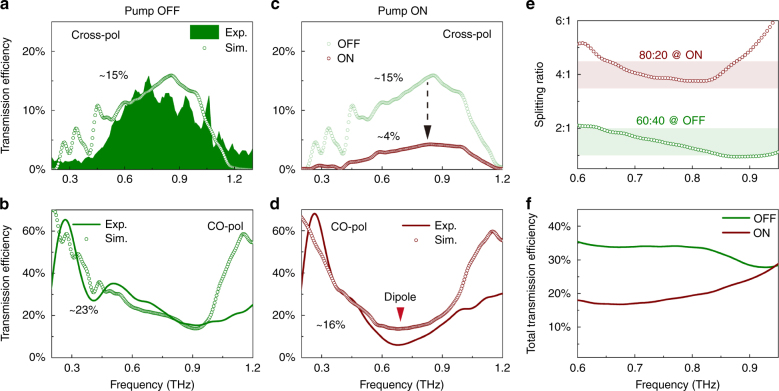
Active control of the beam splitting ratio. **a** Experimental and simulated cross-polarized transmission efficiency in the OFF state, and **b** the correlated co-polarized transmission efficiency. **c** Cross- (simulations in the ON and OFF states for comparison) and **d** co-polarized (experiment and simulation) transmission efficiencies in the ON state. **e** Resultant active modulation of the broadband beam splitting ratio between the co- (normal) and cross-polarized (deflected) components, and **f** the operating efficiency of the active beam splitter

We further measured the active modulation of the beam splitting ratio using the OPTP and fiber-based terahertz time-domain spectroscopic systems. Fig. 5a, b show the measured transmission efficiency of the cross- and co-polarized components in the OFF state, respectively. Since the cross-polarized component is dispersed in the angular space, we plot the transmission efficiency spectra from +0° to +80° with an angular resolution of 1°, as shown by the colored area in Fig. 5a, which also provides a profile of the frequency-dependent intensity spectrum. An overall efficiency of 15% was obtained in a broad bandwidth range extending from 0.6 to 1.0 THz. Such a broadband cross-polarized efficiency originates from the intrinsic anisotropy of the *h*-SRRs, which also enables a broadband multi-mode co-polarized spectrum with the three fundamental modes simultaneously excited for each *h*-SRR unit cell. The co-polarized transmission component is nondispersive in angular space, which allows for the direct measurement of the intensity, as shown in Fig. 5b, where a broadband multi-mode spectrum is observed. The respective simulation results are also shown to verify the measurements. The numerically calculated data agreed well with the measurements except for the slight divergence at very low and high frequencies, which was due to the limitations of our experimental systems (see supplementary Note S7).

In the ON state with an external pump for ultrafast modulation of the beam splitting ratio, the transmission efficiencies were measured in the OPTP system, where a coherent amplified pump beam was introduced for ultrafast optical control. Owing to the stationary transmitter and receiver in the free-space OPTP terahertz spectroscopic system, the modulation of the angle-resolved cross-polarized efficiency could not be directly measured. However, the modulation was readily observed in simulations by adopting the experimental photoconductivity values of a silicon epilayer (*σ* = 15,369 S/m at a 1.9 mJ/cm^2^ pump fluence). As shown in Fig. 5c, the relative  intensity modulation of ~73% is observed for the deflected cross-polarized component. Moreover, we experimentally monitored the ultrafast modulation of the nondispersive co-polarized transmission spectrum transforming from a multimode scenario (Fig. 5b, OFF state) to a pure dipole mode (Fig. 5d, ON state) for a normal transmission. The corresponding simulation results for the ON state reveal a pure dipole mode spectrum that agrees well with the experimental data. These results also validate the rationality of the cross-polarized spectrum modulation due to the correlated nature between the co- and cross-polarized components.

Such an ultrafast correlated modulation of the co- and cross-polarized components could enable an all-optical active polarizing beam splitter with an adjustable ratio, as schematically illustrated in Fig. 4b. The beam splitting ratio is defined as the ratio between the co- (normal) and cross-polarized (deflected) transmission intensities, which reveals a broadband feature, as shown in Fig. 5e, and the ratio is switched from 60:40 to 80:20 for the OFF and ON states, respectively. In fact, the splitting ratio can be continuously modulated within the OFF and ON states by adjusting the pump fluences (see supplementary Figure S8). Note that the total operating efficiency is limited to 40% (Fig. 5f) due to the intrinsic theoretical limitation of single layer metasurfaces^50^ and the high dissipation in metals. However, several feasible approaches can be applied to improve the efficiency, such as introducing multilayer metasurfaces^51^, operating in reflection mode in a sandwich-type configuration^23^ (see supplementary Figure S9) or replacing the metal constituents with dielectric counterparts^52^. Such a beam splitter is ultrathin and miniature with an ultrafast tunable splitting ratio, which could find potential applications in photonic circuits and integrated optical chips for micro-photonic systems.

## Conclusions

In summary, we demonstrated optical switching of the anisotropy of a hybrid metamaterial resonator on a time scale of ~667 ps. This resulted in an ultrafast switching of polarization states with a large polarization contrast. An active polarizing beam splitter with an adjustable beam splitting ratio was also experimentally demonstrated, which is enabled by the tunable dynamic photoconductivity of silicon in the hybrid resonator design. In addition to the discussed prototypes, the applications of our metadevice can be easily extended to designing different metasurface-based active planar optical components, such as ultrafast varifocal planar lenses for photosensitive auto-focus cameras and real-time holographic displays. With the excellent scalability of metamaterials, such a hybrid configuration could be implemented in a broadband spectral regime from the microwave to visible domains. The presented miniaturized ultrathin hybrid metadevices could provide solutions to integrated optical systems with ultrafast data encoding and polarization-division multiplexing for terahertz wireless communications.

## Electronic supplementary material

Supplementary Material for All-optical active THz metasurfaces for ultrafast polarization switching and dynamic beam splitting
