# Supplementary material for: All-optical active THz metasurfaces for ultrafast polarization switching and dynamic beam splitting
Source: Light Sci Appl. 2018 Jul 4;7:28. doi: 10.1038/s41377-018-0024-y (PMC6107012; doi:10.1038/s41377-018-0024-y)
Supplement: Supplementary file 1 — Supplementary Material for All-optical active THz metasurfaces for ultrafast polarization switching and dynamic beam splitting [file 41377_2018_24_MOESM1_ESM.pdf]

Supplementary Material for All-optical active THz metasurfaces for ultrafast polarization switching and dynamic beam splitting

Longqing Cong,[1,2] Yogesh Kumar Srivastava,[1,2] Huifang Zhang,[3] Xueqian Zhang,[3] Jiaguang Han,[3] and Ranjan Singh[1,2,*]

[1]*Division of Physics and Applied Physics, School of Physical and Mathematical Sciences, Nanyang Technological University, 21 Nanyang Link, Singapore 637371, Singapore*

[2]*Centre for Disruptive Photonic Technologies, The Photonics Institute, Nanyang Technological University, 50 Nanyang Avenue, Singapore 639798, Singapore*

[3]*Center for Terahertz Waves and College of Precision Instrument and Optoelectronics Engineering, Key Laboratory of Optoelectronics Information and Technical Science, Ministry of Education, Tianjin University, Tianjin 300072, People's Republic of China*

[*] Email: ranjans@ntu.edu.sg

Supplementary Figures

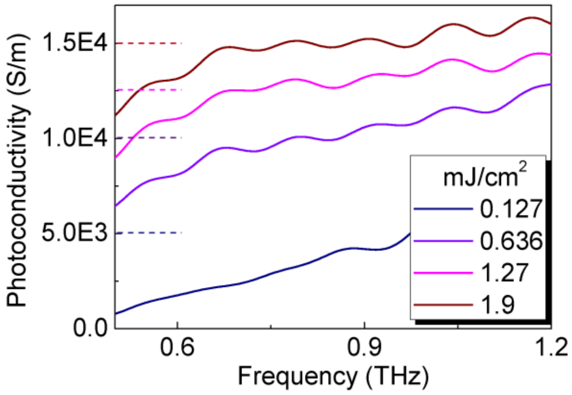

Supplementary Figure S1 Experimentally retrieved frequency-dependent photoconductivity at different pump fluences (see supplementary Note S8).

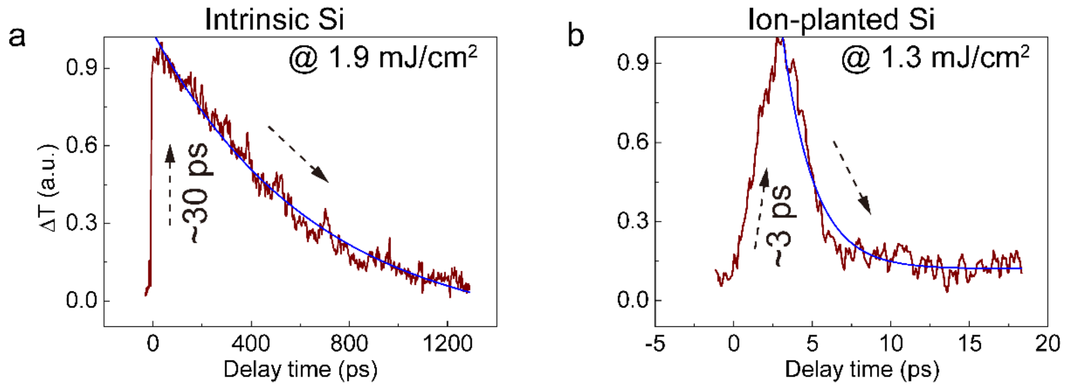

Supplementary Figure S2 Normalized photocarrier dynamics for intrinsic and ion-planted silicon (SOS wafer). The relaxation is fitted by a monoexponential decay equation, and decay constants are determined as 667 ps and 2 ps, respectively. Two orders enhancement of switching time is achieved by Ion-planted approach.

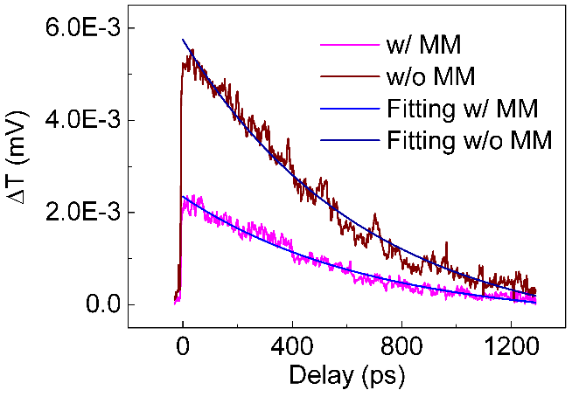

Supplementary Figure S3 Photocarrier dynamics for SOS wafer with (w/) and without (w/o) metasurface (MM) residing on top (not normalized). Identical photocarrier relaxation dynamics is observed, which indicates the switching time of the hybrid metadevice is only determined by the properties of SOS wafer.

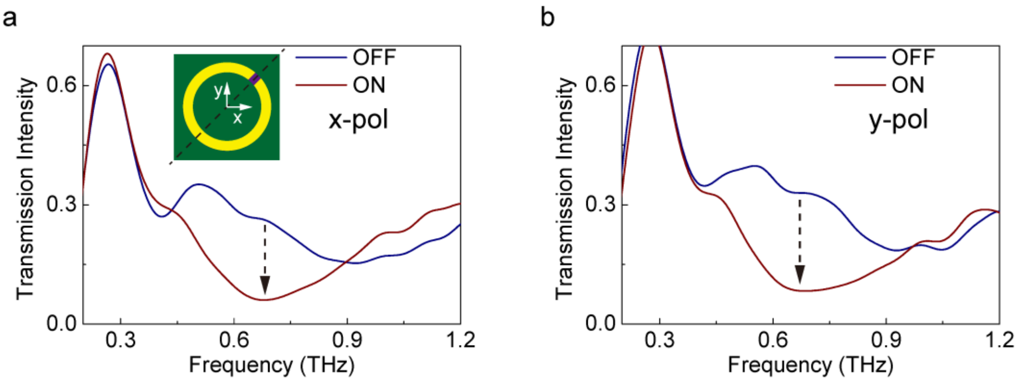

Supplementary Figure S4 Experimental transmission intensity spectra of *h*-SRR metadevice. Co-polarized components (a, T_{xx} and b, T_{yy}) with *x*- and *y*-polarized incidence in the OFF and ON states reveal almost the same response due to symmetry relative to the 45° oriented symmetric axis of the resonator as the dash line indicated in the inset.

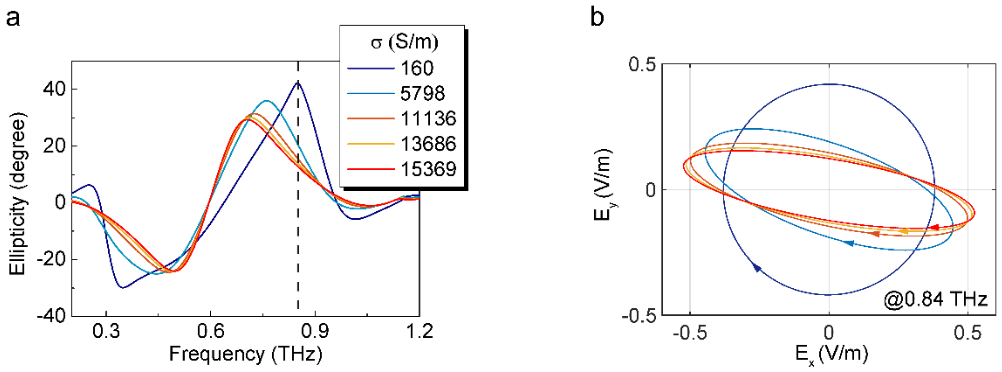

Supplementary Figure S5 Continuous modulation of polarization state by adjusting the pump fluence. (a) Numerical presentation of polarization states in terms of ellipticity. (b) Continuous modulation of output polarization ellipse at 0.84 THz at different pump fluences.

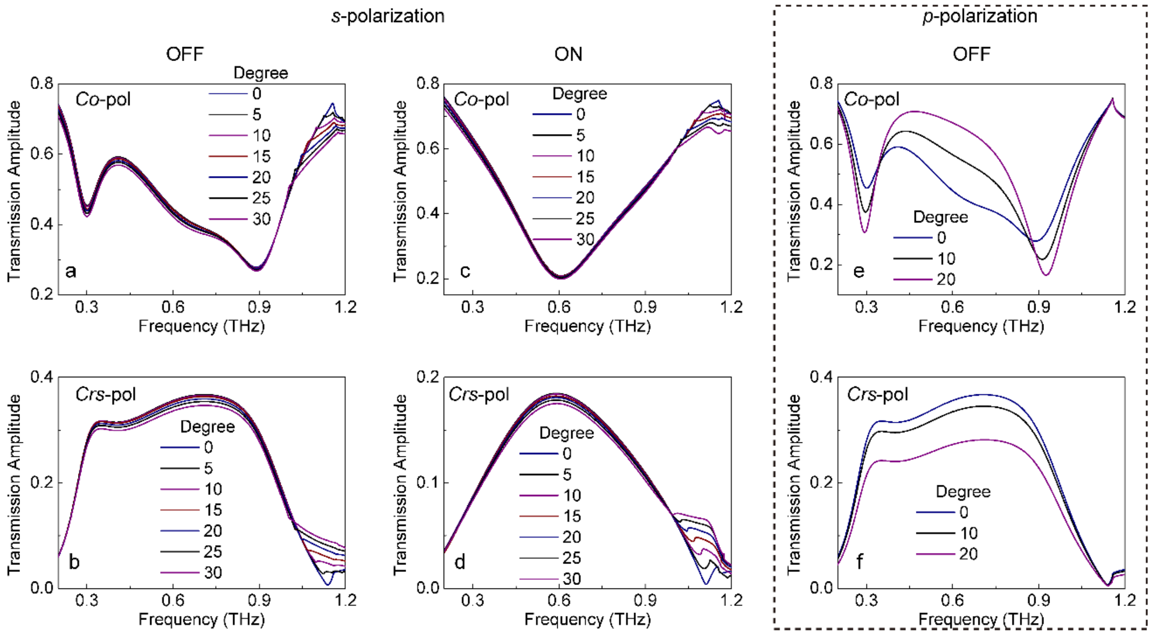

Supplementary Figure S6 Angular dependence of the hybrid metadevice. The angular tolerance tests were performed with *s*- (a-d) and *p*-polarization (e, f) for metadevice in the OFF (a, b, e, f) and ON (c, d) states. The metadevice reveals excellent angular tolerance with *s*-polarization incidence, but reveals strong dependence of *p*-polarization incidence owing to anisotropy of resonators.

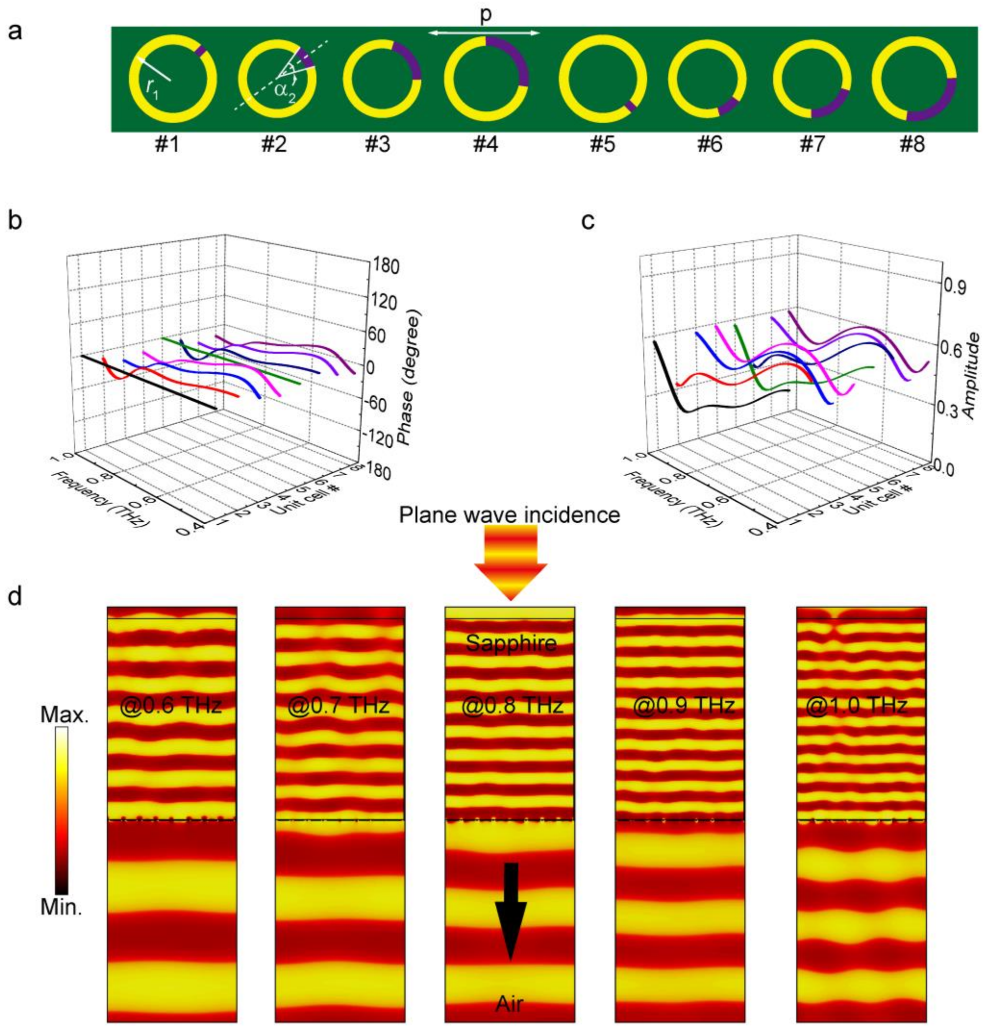

Supplementary Figure S7 Normal transmission of co-polarized component through the dynamic beam splitter. (a) Configuration of one supercell consisting of eight *h*-SRR unit cells for wavefront engineering. (b), (c) Simulated phase and amplitude spectra of co-polarized component of each single *h*-SRR unit cell. (d) Simulated wavefront of co-polarized component with plane wave incidence at different discrete frequencies ranging from 0.6 THz to 1.0 THz.

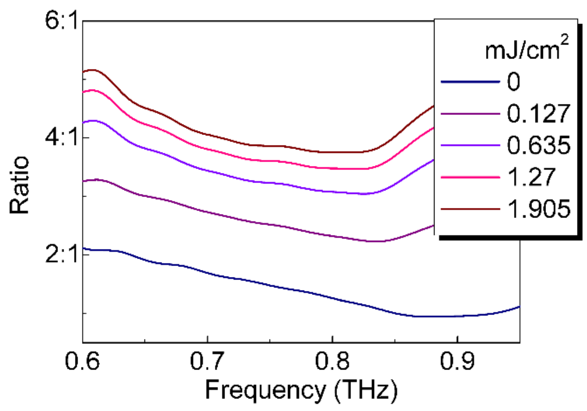

Supplementary Figure S8 Continuous modulation of beam splitting ratio from 60:40 to 80:20 by adjusting the pump fluence from 0 to 1.9 mJ·cm^{-2}.

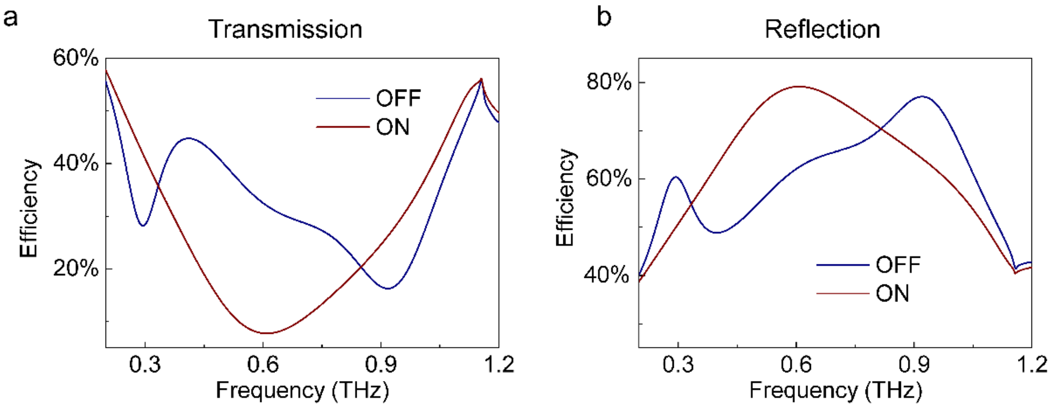

Supplementary Figure S9 Operating efficiency of the hybrid metadevice in transmission and reflection modes in the OFF and ON states. It is observed that the overall efficiency is much improved if the metadevice operates in reflection mode.

Supplementary Table S1 A comparison of active metamaterial schemes

Category	Active media /techniques	Time scale[a]	Stimuli	Area (mm^2)	Modulation	Mode[b]	Comments
Active metasurface applications	GaAs[1]	ms	Electrical	10×10	46%	Broadband	Spectral amplitude modulation
	MEMS[2]	ms	Electrical	10×10	-	Dipole	Multifunctional devices in reflection
	**Silicon on sapphire (SOS, our work)**	**ps**	**Optical**	**10×10**	**85%**	**Broadband**	**Multifunctional devices in transmission**
Single resonance mode modulation	Superconductor (YBCO)[3]	~ps	Optical	10×10	70%	Dipole	Operating @ 20 K
	Silicon on sapphire (SOS)[4]	~ns	Optical	10×10	100%	Fano	Undoped
	GaAs (Gated)[5]	~ms	Electrical	5×5	50%	LC	-
	GaAs (photoexcitation)[6]	~ns	Optical	~10×10	55%	LC	Substrate as active medium
	Liquid crystal[7]	~ms	Electrical	3×3	~75%	Dipole	Operating in reflection
	VO_2[8]	ms	Thermal	-	~10%	LC	-
	MEMS[9]	ms	Electrical	0.1×0.1	56%	Dipole	Operating in reflection@6.2 μm
	Graphene[10]	ns	Electrical	15×15	47%	Dipole	-
	Metal (gold)[11]	fs[12]	Optical	0.1×0.1	-	Fano	Nonlinearity switching
	Perovskite[13]	ns	Optical	10×10	100%	Fano	-

[a]Time scale: estimated dynamic switching time scale. [b]Mode: the resonance mode that is discussed for the dynamic modulation.

Supplementary Notes

Supplementary Note S1: Experimental methods

Sample fabrication. The *h*-SRR samples were fabricated using conventional photolithography combined with reactive ion etching (RIE). Positive photoresist spin-coated on a silicon on sapphire (SOS) wafer was first patterned with the *C*-shape SRR resonator array using a photomask via photolithography, and thermal deposition was adopted to obtain a 200 nm thick aluminum layer on the patterned wafer. After lift-off of the metal, a *C*-shaped metal resonator array was formed on top of the SOS wafer. Then, we patterned the silicon epilayer. A second round of photolithography was performed to define a pattern that covers the split gap section of each SRR resonator with a photomask. With all the circular region under the resonator covered and protected, RIE was used to remove the remaining part of the silicon epilayer.

Simulations. A commercially available software package was utilized to interpret and reproduce the relevant results. Both time-domain and frequency-domain solvers were used with periodic and unit cell boundary conditions, respectively. In simulations, the conductivity of the metal was set with a DC value of $\sigma = 3.72\times10^{7}$ $S\cdot m^{-1}$ in the frequency of interest, and sapphire was considered to be a lossless dielectric with a permittivity of 10.5. The silicon epilayer was modeled as a normal dielectric material with a permittivity of 11.7 and an adjustable conductivity value. The experimentally retrieved DC values of the photoconductivity of the silicon epilayer at different pump

fluences were adopted for the material modeling in simulations in order to accurately reproduce the scenario in the experiments. A plane wave was set as a source in the time-domain solver to model the performance of the active beam splitter.

Angle-resolved measurements. The passive measurement of the angle-resolved spectra was performed using a fiber laser integrated terahertz time-domain system (Menlo system) consisting of a pair of photoconductive antenna transmitter and receiver. The incident terahertz beam was normal to the sample surface, and the transmission signals were recorded by rotating the angle of the receiver around the sample from -80 ° to +80 ° using steps of 1 °. A wire-grid polarizer was inserted in front of the receiver as a polarization analyzer. The time-domain signals were transformed to the frequency domain and normalized to the reference using $\left|\tilde{t}(\omega)\right| = \left|\tilde{E}_S(\omega)/\tilde{E}_R(\omega)\right|$, where $\tilde{E}_S(\omega)$ and $\tilde{E}_R(\omega)$ are the Fourier transformed spectra of the sample and reference, respectively. Note that with the anisotropy of the *r*-cut sapphire substrate, the transmission along the optical axes of the bare SOS substrate was measured as a reference. The transmission intensity was calculated using $T(\omega) = \left|\tilde{t}(\omega)\right|^2$. The measurements were performed at room temperature and in a dry air atmosphere to nullify the effect due to water vapor absorption.

Optical pump-THz probe (OPTP) spectroscopy. The dynamic performance of the hybrid metasurfaces were determined using an optical pump-THz probe (OPTP) spectroscopy setup. A pulsed laser beam from an amplifier (800 nm, pulsed width 120 *f*s, repetition rate 1 kHz, and beam diameter 10 mm) was coherently split into two parts:

an external pump source and a pump beam for generation and detection of terahertz radiation. The external pump beam was not focused in order to cover the entire sample area (10 mm × 10 mm) and guarantee the uniformity of the area probed by the terahertz beam (Φ_t = 3.5 mm at beam waist). For the dynamic terahertz spectrum measurements, the pump beam was delayed by 30 ps (refer to Figure 2c) to capture the maximum modulation of the transmission spectrum, and the pump power (fluence) was adjusted by a continuous nondispersive filter. For the measurements of the free carrier dynamics, we set the delay stage of the terahertz probe to the maximum point of the terahertz time-domain signal and monitored the time-varied terahertz amplitude by adjusting the time instant at which the pump pulse struck the sample by moving the delay stage.

Supplementary Note S2: Saturation of photoconductivity

There will be a saturation of photoconductivity at larger pump fluences, which limits the modulation depth, due to two reasons: 1. Charged carrier density increases and thus carrier-carrier scattering mechanism becomes more pronounced, which reduces the charge carrier mobility and photoconductivity; 2. At high pump fluence, the conduction band will be gradually filled, which shifts the absorption edge according to the Burstein-Moss effect. Therefore, the absorption coefficient of silicon epilayer reduces, which thus limits the increasing of photoconductivity.

Supplementary Note S3: Relaxation dynamics of pure SOS and hybrid metadevices

Relaxation dynamics of silicon epilayer originates from the photoconductivity, which is inversely proportional to the amplitude of transmitted terahertz time-domain signal profile. By mapping the time-dependent variation of the peak amplitude, we can retrieve the photocarrier dynamics. Since the modulation of anisotropy in SRR originates from the photo-induced carriers in silicon epilayer, the relaxation of hybrid metadevice possesses exactly the same time scale (678 ps). The only difference is the absolute change of electric field amplitude (characterized by voltage in THz-TDS) between pure SOS wafer and patterned samples with metasurface. The comparison in the relaxation dynamics of blank SOS substrate and hybrid metadevices is plotted in supplementary Figure S3, where we observe the identical delay constant from the unnormalized relaxation dynamics [$\Delta T = 6.5 \cdot e^{-(t-30)/667} - 0.75$ ($\times 10^{-3}$ mV) and $\Delta T = 2.7 \cdot e^{-(t-30)/678} - 0.35$ ($\times 10^{-3}$ mV)].

Supplementary Note S4: Jones Matrix and the understanding of its elements

In the *h*-SRR metasurfaces, the structures are embedded on substrate with all the materials being linear and reciprocal. With a plane wave propagating in the positive *z* direction, the electric field is described by $E_i(\vec{\mathrm{r}},\mathrm{t}) = \begin{pmatrix} i_x \\ i_y \end{pmatrix} e^{i(\mathrm{kz}-\omega \mathrm{t})}$ where ω is the frequency, $k = \omega / c\sqrt{\varepsilon(\omega)}$ is the wave vector and the complex amplitude i_x and i_y are the states of polarization. The transmission field is described as $E_t(\vec{\mathrm{r}},\mathrm{t}) = \begin{pmatrix} t_x \\ t_y \end{pmatrix} e^{i(\mathrm{kz}-\omega \mathrm{t})}$.

A generalized Jones calculus (*T* matrix) could be adopted for a coherent plane wave, instead of the Mueller calculus that is necessary for incoherent light, to connect the

incidence and transmission. The relation is: $\begin{pmatrix} t_x \\ t_y \end{pmatrix} = \begin{pmatrix} T_{xx} & T_{xy} \\ T_{yx} & T_{yy} \end{pmatrix} \begin{pmatrix} i_x \\ i_y \end{pmatrix} = \hat{T}^f \begin{pmatrix} i_x \\ i_y \end{pmatrix}$, where $\hat{T}^f = \begin{pmatrix} T_{xx} & T_{yx} \\ T_{xy} & T_{yy} \end{pmatrix}$ is the Jones matrix and the superscript f designates the forward propagation.

Due to the reciprocity theorem, the Jones matrix is in the form of $\hat{T}^b = \begin{pmatrix} T_{xx} & -T_{yx} \\ -T_{xy} & T_{yy} \end{pmatrix}$ for the backward propagating wave through the metasurface. On the other hand, the light will see the h-SRR as its mirror image of the structure in the backward propagation scenario. We can then perform a matrix operation to obtain the Jones matrix that describes the metasurface consisting of mirrored h-SRR by using an operator $D = \begin{pmatrix} 1 & 0 \\ 0 & -1 \end{pmatrix}$. The transformed Jones matrix is $\hat{T}^f_{mirror} \equiv D^{-1}\hat{T}^f D = \begin{pmatrix} T_{xx} & -T_{xy} \\ -T_{yx} & T_{yy} \end{pmatrix}$. Since $\hat{T}^f_{mirror}$ and $\hat{T}^b$ describe the same configuration, they should be equal to each other. Finally, we get $T_{xy} = T_{yx}$, which in fact is tenable for all the planar metasurfaces without nonlinear and magnetic materials.

For the co-polarized elements, i.e. T_{xx} and T_{yy}, it could be intuitively understood that the amplitude of them ($|T_{xx}|$, $|T_{yy}|$) should be identical with each other for the h-SRR since the symmetric axis lies along the 45° axis, and the x- and y-polarized incidence will see the resonator in the same fashion except a 180° geometric phase difference. The experimental results of co-polarized transmission intensity with x- and y-polarized incidence are plotted in Supplementary Figure S4 for both OFF and ON states of the h-SRR metasurface. We can observe almost identical responses between the orthogonally

polarized incidence. Therefore, we would only need a pair of elements, i.e. T_{xx} and T_{yx} or T_{yy} and T_{xy}, to describe the transmission properties of the *h*-SRR metasurfaces.

Supplementary Note S5: polarization parameters

The output polarization states are numerically described by Stokes Parameters by using the direct measured elements in Jones matrix (normalized):

$$\begin{aligned} S_0 &= |T_{xx}|^2 + |T_{yx}|^2 \\ S_1 &= |T_{xx}|^2 - |T_{yx}|^2 \\ S_2 &= 2|T_{xx}||T_{yx}|\cos\varphi_{diff} \\ S_3 &= 2|T_{xx}||T_{yx}|\sin\varphi_{diff} \end{aligned},$$

where the first and second subscripts in T_{ii} refer to the transmitted and incident electric field polarization, and $\varphi_{diff} = arg(T_{yx}) - arg(T_{xx})$. The figure of merit of the output polarization states is defined through the ellipticity χ, $\sin 2\chi = \frac{S_3}{S_0}$, where χ = 45° indicates a perfect right-handed circularly polarized light (RCP), χ = -45° indicates a perfect left-handed circularly polarized light (LCP), and χ = 0 indicates the perfect linearly polarized light (LP).

Supplementary Note S6: Normal transmission of co-polarized component

Since the radiation phase of co-polarized component shows independent on the geometrical size as well as the spatial rotation of *h*-SRR, we observe an almost constant phase value around 0° (using phase response of unit cell #1 as reference) as shown in supplementary Figure S7b. The radiation amplitude of each unit cell also reveals a

stable value in a broad frequency band as shown in Figure S7c. Therefore, no extra momentum is introduced for the co-polarized component, and thus the light still propagates in the normal direction in the entire frequency regime of interest as shown in supplementary Figure S7d.

Supplementary Note S7: Experimental limitation

For the experimental characterization, we had to perform measurements by using different terahertz setups. For the passive angle-resolved measurements, we employed the fiber laser integrated terahertz time-domain system whose transmitter and receiver are flexible to be moved in free space. By inserting a wire-grid polarizer in front of receiver, we were able to selectively capture the co- or cross-polarized components by rotating the aligned polarizer at 0° or 90°. The sample was set at the center of rotating circle of receiver, and far-field time-domain transmission signals were measured for angles ranging from -80° to +80° with a step of 1°. The sample might not be accurately resided in the center of the rotating receiver circle, which would introduce systematic errors. Two limitations exist in the fiber laser system: 1. Since the transmitter and receiver are pumped by a fiber coupled oscillation beam, the pulse energy is too low to excite the free carriers in the hybrid metasurfaces. Therefore, experiments of ultrafast dynamics, ultrafast switch of polarization states, and active modulation of beam splitting ratio cannot be done by using this setup. 2. Since the cross-polarized component is dispersed in angular space, we have to collect the frequency-dependent intensity spectrum in an indirect way as the shaded area shown in Figure 5a.

For the active measurements which require a coherent high power source as pump, we have to utilize another terahertz setup, ZnTe crystal based TDS system. The generation of terahertz radiation is based on the nonlinear effect of ZnTe crystal with a high power amplified laser beam in this system. A coherent amplified laser beam was split as a pump source for the dynamic measurements. However, only free-space laser beam was available for this setup, and rigorous alignment was required for the optical setup. Therefore, we could only perform the normal transmission measurements in this stationary system. In this context, it is not possible to capture the direct evidence of cross-polarized intensity modulation for the beam splitter with external pump, but an indirect evidence of the correlated co-polarized intensity modulation was experimentally captured.

Supplementary Note S8: Photoconductivity

The frequency-resolved photoconductivity of unpatterned silicon on sapphire substrate was measured by using the OPTP method at different pump fluences. On the basis of the free carrier dynamics of silicon epilayer as shown in Figure 2c, the pump beam was set ~30 ps ahead of the terahertz pulse so that terahertz pulse captures the maximal accumulation of photo-induced carriers when reaching the sample surface. First, the static transmission spectrum of SOS substrate without pump was measured as a reference (electric field amplitude, $E_0(t)$). Then we moved to the dynamic measurements at different pump fluences, and obtained the respective terahertz time-

domain signals ($E_p(t)$). After Fourier transform, we retrieved the frequency-resolved photoconductivity $\Delta\sigma(\omega)$ according to the equation $\Delta\sigma(\omega)=\frac{\varepsilon_0 c}{d}(n_a+n_b)\frac{-\Delta E(\omega)}{E_0(\omega)}$ where $\Delta E(\omega)$ is the difference between $E_0(\omega)$ and $E_p(\omega)$. Note that the time-domain signal measurements at each pump fluence were repeated for at least three times, and an averaged value was adopted for the photoconductivity retrieve. The influence of noise will be largely excluded, especially for the cases at higher pump fluences where the transmission intensity of terahertz pulse is strongly attenuated.

Supplementary references:

1. Su X, *et al.* Active metasurface terahertz deflector with phase discontinuities. *Opt Express* 2015; **23**: 27152-27158.
2. Cong L, *et al.* Active Multifunctional Microelectromechanical System Metadevices: Applications in Polarization Control, Wavefront Deflection, and Holograms. *Advanced Optical Materials* 2017; **5**: 1600716.
3. Singh R, *et al.* Optical tuning and ultrafast dynamics of high-temperature superconducting terahertz metamaterials. *Nanophotonics* 2012; **1:** 117-123.
4. Gu J, *et al.* Active control of electromagnetically induced transparency analogue in terahertz metamaterials. *Nature communications* 2012; **3**: 1151.
5. Chen HT, Padilla WJ, Zide JM, Gossard AC, Taylor AJ, Averitt RD. Active terahertz metamaterial devices. *Nature* 2006; **444**: 597-600.
6. Padilla WJ, Taylor AJ, Averitt RD. Dynamical Electric and Magnetic Metamaterial Response at Terahertz Frequencies. *Phys Rev Lett* 2006; **96**: 107401.
7. Savo S, Shrekenhamer D, Padilla WJ. Liquid Crystal Metamaterial Absorber Spatial Light Modulator for THz Applications. *Advanced Optical Materials* 2014; **2**: 275-279.
8. Driscoll T, *et al.* Memory metamaterials. *Science* 2009; **325**: 1518-1521.
9. Liu X, Padilla WJ. Dynamic Manipulation of Infrared Radiation with MEMS Metamaterials. *Advanced Optical Materials* 2013; **1**: 559-562.
10. Lee SH, *et al.* Switching terahertz waves with gate-controlled active graphene metamaterials. *Nat Mater* 2012; **11**: 936-941.
11. Ren M, Plum E, Xu J, Zheludev NI. Giant nonlinear optical activity in a plasmonic metamaterial. *Nature Communications* 2012; **3**: 833.
12. Ren M, *et al.* Nanostructured Plasmonic Medium for Terahertz Bandwidth All-Optical Switching. *Adv Mater* 2011; **23**: 5540-5544.
13. Cong L, Srivastava YK, Solanki A, Sum TC, Singh R. Perovskite as a Platform for Active Flexible Metaphotonic Devices. *ACS Photonics* 2017; **4**: 1595-1601.
